# Increased belongingness as a mechanism of change of school-based programs mitigating suicidal ideation among adolescents

**DOI:** 10.1007/s00787-025-02915-2

**Published:** 2025-11-26

**Authors:** Shira Barzilay, Daniella Ekstein, Itay Cohen, Nofar Stein, Keren Tal Von Strauss, Mira Levis Frenk

**Affiliations:** 1 Department of Community Mental Health, University of Haifa, Haifa, Israel; 2 Department of Psychological Medicine, Schneider Children’s Medical Center, Petach Tikva, Israel; 3 Ruth and Bruce Rappaport Faculty of Medicine, Technion – Israel Institute of Technology, Haifa, Israel; 4 Department of Psychology, College of Management, Rishon LeZion, Israel

**Keywords:** Suicide, School-based intervention, Adolescents, Belongingness, IPTS

## Abstract

Suicide is a leading cause of death among adolescents, highlighting the importance of early intervention in school settings. Universal mental health awareness programs, aimed at improving mental health literacy and help-seeking behaviors, are a key preventive approach. This study examined whether changes in thwarted belongingness and perceived burdensomeness—two constructs from the Interpersonal Theory of Suicide (IPTS)—mediate the effects of school-based mental health interventions on suicidal ideation (SI). A total of 436 adolescents from central Israel (154 boys, 279 girls; M = 14.6, SD = 1.1) were randomly assigned to either a mental health awareness intervention (*N* = 256) or a minimal-intervention control group with attendance monitoring (*N* = 180). Belongingness, burdensomeness, and SI were assessed at baseline, 1-month, and 1-year post-intervention. Regression analyses showed that lower belongingness (β = –0.25, *p* < .001) and higher burdensomeness (β = 0.21, *p* < .01) predicted higher SI at 1-month, while only belongingness remained significant at 1-year (β = –0.19, *p* < .01). The mental health awareness intervention increased school-specific belongingness (β = 0.22, *p* < .001), whereas attendance monitoring intervention increased general belongingness (β = 0.18, *p* < .01). Mediation analysis indicated that the effects of the intervention type on SI were not significantly mediated by general and school belongingness. These findings suggest that promoting broader social connectedness beyond school environments may support reductions in adolescent suicide risk over time.

## Introduction

Suicide is a phenomenon that transcends continents, religions, and social groups [1]. According to the World Health Organization, suicide is one of the leading causes of death globally and is one of the three most common causes of death among youth and adolescents [2]. Moreover, there has been a clear upward trend in the number of adolescents seeking emergency room treatment due to suicidal ideation (SI) and behaviors [3].

Adolescence is characterized by significant changes across various areas of an individual’s life [4]. During this key developmental stage, identity formation, emotion regulation, and peer relationships become central, making adolescents vulnerable to mental health challenges. There is an increased sensitivity to social belonging alongside evolving coping mechanisms. As a result, adolescence is a key period for intervention, specifically for targeted prevention efforts in reducing risk. In the effort to trace risk factors that may lead adolescents to experience SI and behaviors, various factors have been identified [5]. One risk factor that is strongly linked to adolescent suicide, specifically within a school setting, is a lack of belongingness. Belongingness is the fundamental need to feel connected, accepted, and valued within social relationships and groups [6]. In a school setting, belongingness can refer to a sense of being accepted within one’s school community. This includes positive peer relationships and supportive interactions with teachers [7]. Research has shown a relationship between a supportive school environment and reduced SI and behaviors, emphasizing the impact of the school setting on levels of SI [8]. Other studies have demonstrated that enhancing a sense of belonging is associated with a decrease in SI among school students, as belongingness is considered essential for human connection and well-being [9].

The role of school belongingness can be understood through the lens of the Interpersonal Theory of Suicide (IPTS) proposed by Joiner [10]. This theory suggests that low *belongingness* can contribute to SI. Low belongingness is a sense of loneliness, rejection, and lack of reciprocal care [11] often stemming from unmet relational needs [12]. Studies on adolescents have shown that low belongingness is associated with SI [13] and, conversely, when adolescents experience an increased sense of belonging, SI decreases [14]. The term *perceived burdensomeness* is also deemed a central factor linked to SI according to the IPTS [15]. Meaning, the stronger the perception of being a burden, the more frequent and intense the SI [16].

Evidence from community samples on the role of belongingness and perceived burdensomeness in SI is mixed [17]. Some studies found that both thwarted belongingness and perceived burdensomeness are associated with SI [18–20]. Others reported that perceived burdensomeness was more strongly linked to past and future SI than thwarted belongingness [21–24], while some studies identified thwarted belongingness as the key predictor [25, 26]. These findings underscore the complex and variable roles of perceived burdensomeness and thwarted belongingness in community settings. To our knowledge, no study has examined the effect of school-based universal suicide prevention programs on belongingness and perceived burdensomeness as potential mechanisms for mitigating SI.

### School-based suicide prevention programs

The importance of a strong sense of belongingness is evident in its integration into various suicide prevention programs. Adolescents develop this sense of belongingness when they feel supported and valued by those in their environment [27]. School-based mental health and suicide prevention education programs foster belongingness by promoting empathy and sensitivity within the school community [28]. Another approach involves training “gatekeepers” to create environments that facilitate help-seeking and enhance connectedness [29]. Such educational and skill-building initiatives shift the way mental illness and suicide are addressed, fostering resilience factors that can reduce suicide risk [30].

One widely implemented school-based prevention program is “Youth Aware of Mental Health” (YAM). This intervention program combines cognitive and emotional strategies to help adolescents internalize the complexities of mental health [31]. YAM incorporates role-playing and discussions, allowing participants to navigate dilemmas, explore coping strategies, and engage in peer support. Rather than providing a predetermined skill set, facilitators encourage adolescents to experiment, share perspectives, and develop their own approaches to challenges. These activities create an interactive space that directly promotes help-seeking [32]. YAM has been associated with a reduction in SI and behaviors among adolescents across ten European countries [33]. Research suggests that program participation alters adolescents’ coping styles during times of crisis, contributing to fewer SI [34]. However, less is known about how suicide prevention universal school-based programs influence belongingness as a protective factor against SI among adolescents.

The current study therefore aims to (1) examine the prospective predictions of the IPTS constructs *belongingness* and *burdensomeness* in a community sample of adolescents, and (2) assess whether participation in universal school-based suicide prevention programs increases adolescents’ general and school-specific sense of belongingness and whether this increase, in turn, affects the level of SI. We hypothesized that both belongingness and burdensomeness would predict SI, and that the mental-health awareness program would enhance belongingness, mediating the reduction in SI among adolescents.

## Methods

### Sample description

The sample consisted of 436 students who were randomly assigned to either a universal mental health awareness suicide prevention intervention group (*N* = 256) or a minimal intervention (control) group, including attendance monitoring (*N* = 180). Participants included 154 boys and 279 girls aged 12–18 (M = 14.6, SD = 1.1), who attended high schools in a central Israeli city. The majority of participants were born in Israel (78%), while the remaining participants (22%) identified as immigrants. It is important to note that immigration status was assessed, as suicide rates among immigrants from the former Soviet Union and Ethiopia are historically higher. Over recent years, these rates have declined [33]. However, it remains a relevant factor to consider when evaluating those of immigrant status in the country. Schools were randomly selected and assigned to one of two intervention types. Statistically significant age differences were found, with the mental health awareness intervention group having a higher average age (14.9) compared to the minimal intervention group (14.07). Gender differences were also significant, as the intervention group included a higher proportion of boys (60.8%), while the minimal intervention group included only girls. Considering this, gender was included as a control variable in data analysis.

## Measures

### Interpersonal needs questionnaire (INQ)

This validated self-report questionnaire examines variables of the interpersonal theory of suicide [35]. The shortened version includes 12 items; seven measure perceived burdensomeness (e.g., “These days, I feel that I have disappointed the people in my life”) and five measure belongingness (e.g., “These days, other people care about me”). Responses are rated on a scale from 1 (not true at all) to 7 (very true). Higher scores indicate greater perceived burdensomeness or lower belongingness. Good internal consistency reliability has been reported, with alpha coefficients of 0.89 for burdensomeness and 0.85 for belongingness [36]. This study’s reliability ranged from 0.82 to 0.76 for burdensomeness and 0.92 to 0.97 for belongingness across three measurement points.

### School climate questionnaire

This self-report measure assesses school-specific belongingness [37]. Participants rate their agreement with statements about their school experiences on a scale from 1 (never) to 6 (daily) (e.g., “The teachers care about me as a person”). Higher scores indicate a stronger sense of school belonging. Reliability in this study ranged from 0.81 to 0.91.

### Paykel suicide scale

This self-report scale assesses levels of SI [38]. Participants indicate whether they have experienced specific thoughts over the past year or the past two weeks. The scale comprises five items, each rated from 0 (never) to 5 (always). Previous studies have reported good internal consistency reliability (α = 0.85). In this study, reliability ranged from 0.87 to 0.94.

### Procedure

This study was part of an adolescent health promotion initiative conducted in schools across Israel. The Helsinki Committee of the Rabin Medical Center, the Israeli Ministry of Health, and the Ministry of Education’s Chief Scientist approved the research. Parents and adolescents provided informed consent after receiving detailed explanations about the research objectives and procedures.

Participants completed self-report questionnaires within 60–90 min during school hours. The questionnaires were administered by a research assistant and a teacher. Research assistants attended the sessions to explain and support in case of difficulties, while teachers addressed broader logistical issues. The questionnaires were distributed three times over one calendar year: at baseline assessment, at a one-month follow-up, and at a 12-month follow-up.

### Description of interventions

#### Universal mental-health awareness intervention group

The program was adapted for Israeli youth based on existing mental health intervention programs [32] designed to promote mental health awareness and resilience among students. The program includes three hours of role-playing sessions, an interactive workshop supplemented by a 32-page booklet for students to take home, and six educational posters displayed in classrooms. Additionally, it features two one-hour lectures on mental health topics, delivered at the beginning and end of the intervention. Trained facilitators, using a structured 31-page methodology guide, implemented the program to ensure consistency and effectiveness in delivery.

#### Minimal intervention group

Ethical considerations prevented the use of a purely non-intervention control group. Instead, the minimal intervention group was exposed to six educational posters identical to those in the intervention group, providing contact information for local health services. Additionally, students’ attendance was monitored throughout the study, and parents were informed about their child’s presence or absence (Table 1).

**Table 1:** Correlations between changes in belongingness, burdensomeness, and suicidal ideation (SI) (1-month and 1-year follow-ups)

Follow-up time	Variables	SI	Belongingness	Perceived burdensomeness
1-Month	SI	—	−0.27**	0.44**
	Belongingness		—	−0.39**
	Perceived Burdensomeness			—
1-Year	SI	—	−0.34**	0.21**
	Belongingness		—	−0.37**
	Perceived Burdensomeness			—

*p* <.001 for all correlations

#### Statistical analyses

Associations between changes in school belonging, burdensomeness, and SI were examined using Pearson correlations. Linear regression analyses were conducted to predict changes in SI based on changes in belongingness and burdensomeness, controlling for gender. The effect of the intervention program on general and school-specific belonging was analyzed using two-way repeated-measures ANOVAs, with gender as a control variable. Finally, mediation models were tested to examine whether increases in belonging mediated the relationship between participation in the intervention program and reductions in SI, controlling for gender (Table 2).

**Table 2:** Means and standard deviations of general belongingness, school belongingness, and suicidal ideation (SI) over time by group

Timepoint	Group	General belongingness			School belongingness			Suicidal ideation		
		N	M	SD	N	M	SD	N	M	SD
Baseline	Intervention	169	28.14	7.19	175	2.77	0.66	190	1.56	3.22
Baseline	Minimal Intervention	95	26.6	8.79	111	2.96	0.70	120	1.52	3.31
1 Month	Intervention	169	27.97	7.82	175	13.94	3.09	190	0.82	2.54
1 Month	Minimal Intervention	95	28.8	8.23	111	14.64	3.01	120	0.63	1.71
1 Year	Intervention	169	26.20	9.16	175	13.79	3.31	190	1.15	3.05
1 Year	Minimal Intervention	95	29.25	7.16	111	14.74	3.27	120	0.46	1.40

*M* mean, *SD* standard deviation

## Results

**Fig. 1:**
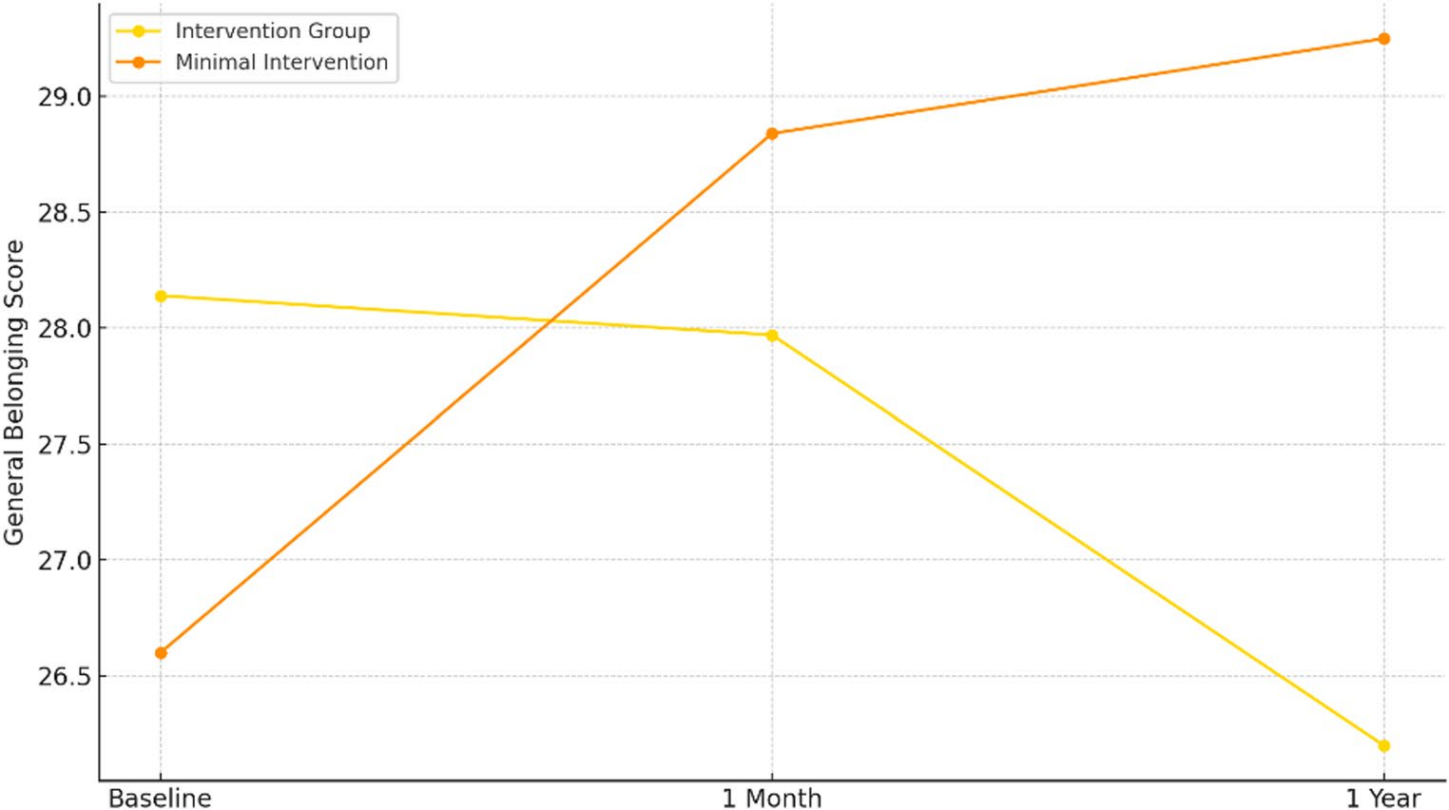
Change in general belongingness over one year by intervention group

Pearson correlation analyses were conducted within the minimal intervention group at two different time points to test whether changes in perceived belongingness and burdensomeness predicted changes in SI. At the one-month follow-up, a significant negative correlation was found between changes in belongingness and changes in SI (*r* = −.27, *p* <.001), and a significant positive correlation was observed between changes in burdensomeness and SI (*r* =.44, *p* <.001). Similar patterns emerged at one year. A negative correlation was again observed between changes in belongingness and SI (*r* = −.34, *p* <.001), and a positive correlation was found between changes in burdensomeness and SI (*r* =.21, *p* <.001) (Fig. 1).

Two multiple linear regressions were conducted to examine further the predictive value of changes in perceived belongingness and burdensomeness. At the one-month follow-up, the overall model significantly predicted changes in SI, F(2, 94) = 16.14, *p* <.001, explaining 25.6% of the variance. Specifically, a decrease in belongingness significantly predicted an increase in SI, β = −0.10, SE = 0.04, β = −0.25, t = − 2.53, *p* <.05. Conversely, an increase in perceived burdensomeness was also a significant predictor of increased SI, β = 0.22, SE = 0.06, β = 0.35, t = 3.57, *p* <.01. A year after the intervention, the regression model was also significant, F(2, 94) = 9.37, *p* <.001, accounting for 16.6% of the variance in SI. Belongingness continued to show a strong predictive relationship with SI: lower feelings of belongingness were associated with higher SI, B = − 0.13, SE = 0.04, β = −0.38, t = − 3.71, *p* <.01. However, perceived burdensomeness was no longer a significant predictor at this timepoint (β = 0.08, SE = 0.12, β = 0.07, t = 0.68).

### The effect of intervention type on belongingness and SI

To test the second hypothesis, repeated-measures ANOVAs were conducted to examine the effects of the intervention on general belongingness, school belongingness, and SI across three different time points: baseline, one-month post-intervention, and one year later.

For general belongingness, results showed no significant main effect of time, F(1, 262) = 0.07, n.s, and no significant main effect of group (F(1, 262) = 0.37, n.s). However, a significant time × group interaction was found (F(1, 262) = 9.71, *p* <.05), indicating differential change over time between the groups. Specifically, the minimal intervention group showed increased general belongingness over time, while the mental health awareness intervention group showed a slight decrease. In contrast, a significant main effect of time was found for school belongingness (F(1, 284) = 167.43, *p* <.001), with no significant main effect of group or interaction effect. These results suggest that both groups improved school belongingness similarly over time. There was a significant main effect of the group for SI (F(1, 308) = 7.98, *p* <.005), with higher SI in the mental health awareness intervention group. No significant interaction or time effect was found, suggesting that although scores changed over time, the pattern was consistent across groups.

### Belongingness mediating the intervention effect on suicidal ideation

Two mediation models were tested to determine whether general and school belongingness mediated the relationship between intervention type and SI. The first model included a one-month follow-up after the intervention, and the second model included a one-year follow-up.

In the one-month follow-up model, no significant total or direct effect of the intervention on SI was found (Total effect estimate = − 0.02, n.s., Direct effect estimate = 0.01, CI = [−1.02, 1.05]). Participation in the mental health awareness intervention significantly predicted general belongingness (β = 0.25, *p* <.01), and general belongingness was a significant predictor of SI (β = −0.12, *p* =.01). The indirect effect did not reach statistical significance (via General Belongingness (estimate=−0.14, CI = [− 0.42, 0.01]), via School Belongingness (estimate = 0.01, CI = [− 0.12, 0.15]).

**Fig. 2:**
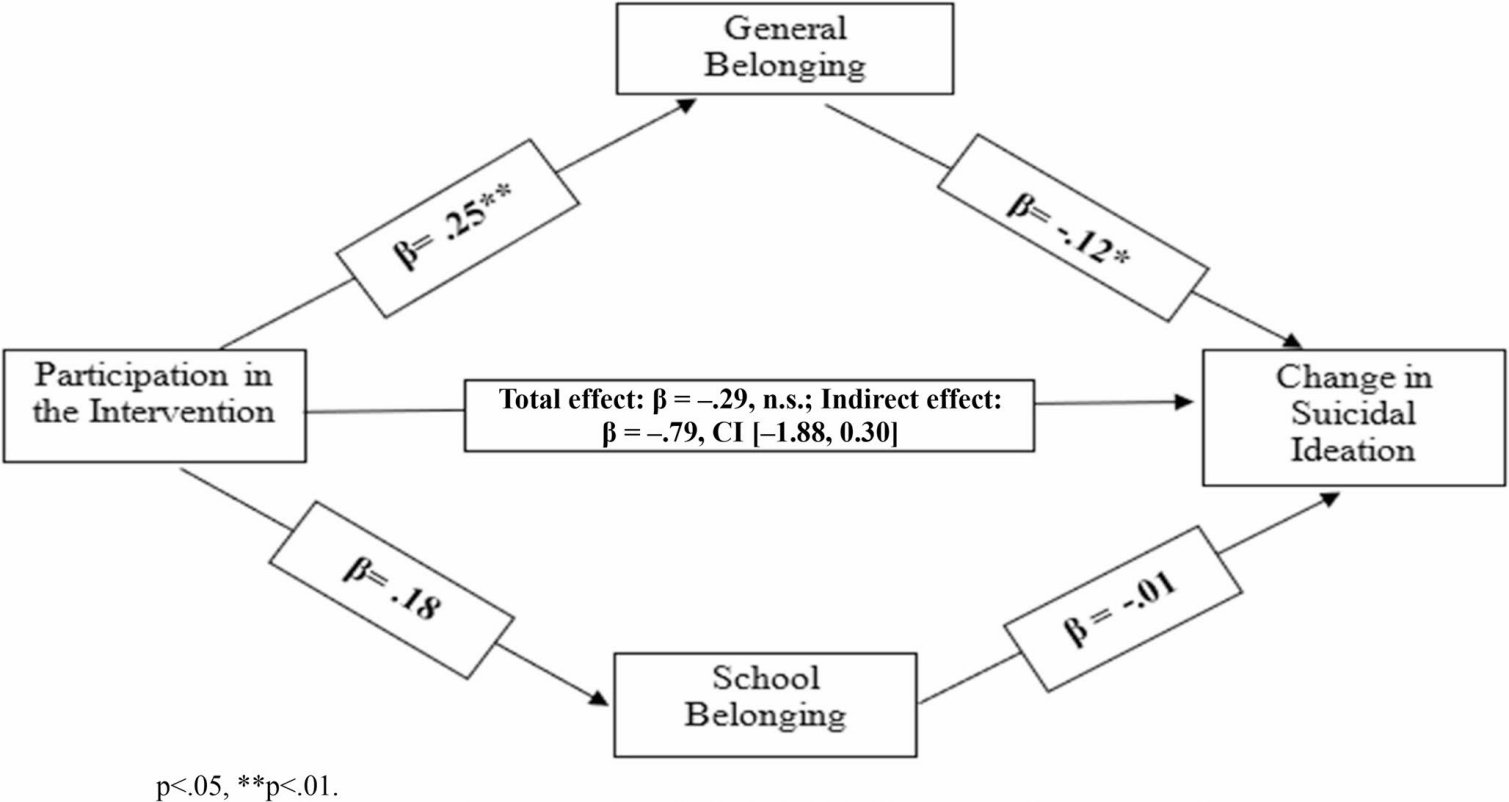
The relationship between participation in the intervention and changes in suicidal thoughts after one year

The one-year follow-up model showed mental health awareness intervention’s total and direct effects on SI were insignificant (total effect β = −0.29, n.s; indirect effect estimate = estimate=−0.79, CI = [− 1.88, 0.30]). Participation in the mental health awareness intervention significantly predicted general belongingness (β = 0.41, *p* <.01), which in turn significantly predicted reduced SI (β = −0.12, *p* <.05). The indirect effect, however, did not reach statistical significance (General Belongingness: estimate=−0.20, CI = [− 0.58, 0.03]; School Belongingness: estimate=−0.08, CI = [− 0.42, 0.10]), and thus the mediation hypothesis was not confirmed (Fig. 2).

## Discussion

This study aimed to examine two key objectives. First, to evaluate the role of changes over time in belongingness and perceived burdensomeness in predicting SI in a community sample of adolescents. Second, to assess whether participation in a school-based universal suicide prevention program enhances general and school-specific belonging, which in turn mediates the program’s effect on SI. The findings supported the first aim hypothesis, which aligns with previous research. In contrast, the second aim yielded mixed and surprising results, contradicting some prior assumptions.

### IPTS predictions

The findings supported the hypotheses of the IPTS, providing additional evidence that both decreased belongingness and increased perceived burdensomeness predict higher levels of SI. However, there was a difference in the prediction over time. While both belongingness and burdensomeness predicted SI in one month, only belongingness was associated with SI one year later. These results contribute to the mixed findings on the specific associations between belongingness, burdensomeness, and SI reported in previous studies of community adolescent samples [17]. The difference found across samples may be attributed to specific measurements, follow-up times, and varying sociodemographic characteristics of the samples and school settings, such that the IPTS variables may play a different role. The current study suggests that belongingness may play a longer-term role in the development of SI among adolescents than burdensomeness. The findings emphasize the vital role of general and school belongingness in SI for adolescents, reinforcing previous studies that highlight belongingness as a protective factor [13, 14]. It is possible that the minimal intervention functioned as an indirect social signal by enhancing accountability and reinforcing adolescents’ perception of being recognized both at school and at home.

### Belongingness as a mechanism of change in school-based universal suicide prevention programs

To our knowledge, this is the first study to examine belongingness as a mechanism of change for school-based suicide prevention and intervention. The results partially supported this mechanism but revealed unexpected patterns. We hypothesized that the mental health awareness intervention would increase belongingness, leading to a reduction in SI. However, the findings suggest a more nuanced relationship. While the mental health awareness intervention increased school belongingness, a minimal intervention, which included attendance monitoring, was associated with increased general belongingness. This suggests that different interventions may influence distinct aspects of belongingness, which may shape suicide risk trajectories in different ways. One interpretation is that the mechanism behind increased general belongingness stems from direct contact between schools and parents. Attendance monitoring required consistent parental updates, which not only engaged caregivers but also reinforced their involvement in the program. This parental engagement may have strengthened adolescents’ perception of being supported at home, highlighting the importance of the family system in tandem with the school system (Bronfenbrenner, 1977). Interventions that deliberately foster this inter-systemic relationship may produce stronger and more sustainable improvements in belongingness.

Prior research supports the notion that school-based interventions can enhance students’ connection to their educational environment [31, 36, 39]. However, interventions focusing solely on the school setting may not sufficiently address broader social connectedness, shaped by familial and community relationships [21, 41]. The present findings suggest that while the mental health awareness intervention effectively fostered school belonging, potentially through peer discussions and role-playing activities, attendance monitoring may have strengthened adolescents’ general sense of belonging by reinforcing parent-adolescent communication and perceived support at home. This aligns with existing research emphasizing the role of parental involvement in strengthening a sense of belonging [12].

These findings suggest that attendance monitoring through structured parental updates functioned as an active intervention by enhancing broader social connectedness. Attendance monitoring may have promoted belongingness in a way that extended beyond a school environment, which differs from interventions that rely on structured school-based interactions. The direct communication between schools and parents may have reinforced the adolescent’s perception of being noticed and valued, ultimately contributing to a greater sense of general belongingness. This may explain why general belongingness, compared to school belongingness, emerged as a significant predictor of reduced SI in both the short and long term. These findings emphasize prior support for enhancing social integration to mitigate suicide risk [40].

Although the reduction in SI was not observed through the expected pathway of belongingness, findings highlight that the intervention group’s impact on SI may be mediated through alternative mechanisms [33], such as improvements in coping strategies, willingness to seek help, reduced stigma of mental health, and stronger peer or teacher support. These factors may play a role in reducing SI independent of belongingness. While belongingness appears to play a role in reducing SI, it may need to be reinforced with additional components to be effective (e.g., emotional resilience training and family involvement) [18]. Future studies should explore whether integrating digital engagement strategies such as structured check-ins, app-based attendance tracking, or automated supportive messages, can enhance the long-term effects of school-based interventions. These approaches could combine the benefits of school-based psychoeducation with ongoing digital monitoring to provide a more comprehensive strategy for suicide prevention in adolescents.

From an implementation perspective, the interventions differed in the demands they placed on schools. The mental health awareness program required trained facilitators, multiple structured activities, and designated instructional time, whereas the minimal intervention was relatively straightforward to implement. These contrasts highlight important considerations for feasibility and may inform decisions about how schools balance resource requirements with anticipated benefits.

Overall, findings suggest that school-based programs may contribute to reducing SI in adolescents, but their effects likely depend on broader social and familial contexts. Future research should continue to examine how different forms of connectedness, combined with complementary supports can sustain long term reductions in suicide risk.

## Limitations

This study has several limitations. The imbalance in gender between groups is an important limitation, as differences in SI and belonginess can vary significantly among boys and girls. Future research should aim to recruit balanced samples so that potential differences in how boys and girls experience belongingness and SI can be examined more clearly. Another limitation is that belonginess did not significantly mediate the intervention effects on SI. This restricts conclusions about belongingness as the primary mechanism of change. Furthermore, each school implemented only one type of intervention, which may have introduced institutional biases and limited the generalizability of the findings. Finally, the reliance on self-report questionnaires introduces potential biases, including social desirability and recall inaccuracies.

### Implications and future directions

These findings suggest that while school-based interventions can enhance belongingness within the educational context, they may not be sufficient to foster a more generalized sense of belonging that extends to family and community connections. Future programs could combine school-based activities with structured family and community aspects. For example, parent-school communication, family workshops, or peer mentoring to strengthen overall belongingness [18]. Additionally, integrating emotional regulation training may enhance the program’s impact on SI. While expanding interventions to include family dynamics is one approach, future adaptations of existing school-based programs, could also integrate structured collaboration between schools and families. Enhancing school-student-family engagement could maximize the strengths of both models while ensuring sustainability within the school setting. Future research should explore multi-layered interventions that combine school, family, and peer support to create a more comprehensive approach to suicide prevention. Longitudinal studies assessing the sustained effects of such interventions would provide valuable insights into their long-term efficacy. Importantly, findings suggest that even minimal efforts to monitor student attendance and inform parents can have significant protective effects. Simply being present in a school environment or knowing that absences are noted may reduce suicidal risks. By enhancing belonging through school-based and family-involved strategies, education systems can provide a cost-effective and impactful approach to addressing adolescent suicide.

## Conclusions

This study supports the role of belongingness as a protective factor against SI but suggests that school-based interventions alone may not be sufficient to enhance general belonging. The school-based universal suicide prevention program effectively increased school belonging, yet broader interventions integrating family and community support may strengthen the overall impact. Future research should refine intervention strategies to maximize the protective benefits of belongingness in adolescent suicide prevention efforts.

## Data Availability

No datasets were generated or analysed during the current study.
